# The effect of selective internal radiation therapy with yttrium-90 resin microspheres on lung carbon monoxide diffusion capacity

**DOI:** 10.1186/s13550-017-0353-5

**Published:** 2017-12-29

**Authors:** Tunc Ones, Emel Eryuksel, Feyyaz Baltacioglu, Berrin Ceyhan, Tanju Yusuf Erdil

**Affiliations:** 1 Department of Nuclear Medicine, Pendik Research and Training Hospital, Marmara University, Istanbul, Turkey; 2 Department of Pulmonary and Critical Care, Pendik Research and Training Hospital, Marmara University, Istanbul, Turkey; 3 Department of Radiology, Pendik Research and Training Hospital, Marmara University, Istanbul, Turkey

**Keywords:** Selective internal radiation therapy (SIRT), Lung carbon monoxide diffusion capacity (DLCO), Radiation pneumonitis

## Abstract

**Background:**

Selective internal radiation therapy (SIRT) with embolization of branches of the hepatic artery is a valuable therapeutic tool for patients with hepatic malignancies; however, it is also associated with lung injury risk due to shunting. Diffusion capacity of the lungs for carbon monoxide (DLCO) is a clinically significant lung function test, and worsening in DLCO is suggested to reflect a limited gas exchange reserve caused by the potential toxicity of chemoradiotherapy or it may be a marker of related lung injury. This study aimed to examine the changes in DLCO during SIRT with resin microspheres in newly treated and retreated patients. Forty consecutive patients who received SIRT for a variety of malignant conditions were included. All subjects were treated with Yttrium-90 labelled resin microspheres. DLCO tests were performed after the procedures. In addition, patients were specifically followed for radiation pneumonitis.

**Results:**

The mean DLCO did not significantly change after the first (82.8 ± 19.4 vs. 83.1 ± 20.9, *p* = 0.921) and the second treatments (87.4 ± 19.7 vs. 88.6 ± 23.2, *p* = 0.256). Proportion of patients with impaired DLCO at baseline was not altered significantly after the first (37.5 vs. 45.0%, *p* = 0.581) and the second treatments (27.3 vs. 27.3%, *p* = 1.000). Also, percent change in DLCO values did not correlate with radiation dose, lung shunt fraction, or lung exposure dose (*p* > 0.05 for all comparisons). None of the patients developed radiation pneumonitis.

**Conclusions:**

Our results suggest that no significant change in DLCO in association with SIRT occurs, both after the first or the second treatment sessions. Further larger studies possibly with different protocols are warranted to better delineate DLCO changes after SIRT in a larger spectrum of patients.

## Background

Selective internal radiation therapy (SIRT), in which the branches of the hepatic artery are embolized using biocompatible Yttrium-90 (^90^Y) labeled microspheres, have recently emerged as an important therapeutic tool for patients with hepatic malignancy. However, after intraarterial injection of ^90^Y-labeled microspheres into the liver, a substantial portion of the radioactive material is shunted into the lung via intrametastatic/intratumoral arteriovenous shunts [1]. When proportion of the shunting of radionuclide microspheres exceeds 15%, the risk of radiation induced pneumonitis (RP) is significantly elevated [2]. It is recommended that SIRT is relatively contraindicated in patients with lung shunting such that the single treatment lung dose will exceed 30 Gy or that the cumulative lung dose will exceed 50 Gy [3, 4].

Diffusion capacity of the lungs for carbon monoxide (DLCO), a clinically important measure of the lung function, gauges the ability of the lungs to transfer the gas in inhaled air to the red blood cells in pulmonary capillaries [5]. General areas of use include the identification of the cause of dyspnea or hypoxemia, monitor the progression of interstitial lung disease, and to detect the presence of pulmonary hypertension in patients under risk [6]. In addition, DLCO may also show the presence of limited gas exchange reserve caused by the potential toxicity of chemoradiotherapy, and previous studies have suggested that the most consistent changes in pulmonary function tests after external beam radiotherapy (RT) are recorded with DLCO [7–9].

Radiation pneumonitis is a rare but serious complication of SIRT that may develop 1 to 6 months after treatment. Further radiation exposure in lungs is not recommended within the first 6 months after SIRT, and in cases where the second SIRT is performed within 6 to 12 months of initial treatment, the recommended dose is < 50% of the maximum tolerated dose that was administered during the initial procedure [10, 11].

Also, DLCO may be a sensitive marker of lung injury, despite the reduction in DLCO is usually subclinical [9, 12–15]. Published data on the degree of radiation-related changes in DLCO after SIRT is limited, and the importance of subclinical lung injury in these patients remains unknown. Nevertheless, DLCO has been recommended as part of the general work-up of such cases [11].

This study aimed to examine the changes in DLCO during SIRT with resin microspheres in newly treated and retreated patients.

## Methods

### Study subjects

Forty consecutive patients who underwent SIRT at the Department of Nuclear Medicine between March 2015 and February 2017 for a variety of malignant conditions involving the liver were prospectively analyzed. The study protocol was approved by the institutional ethics committee (dated, September 2016; No. 09.2016.512).

### Pretreatment evaluation and patient selection

To be eligible for study, patients had to be at least 18 years old, have an Eastern Cooperative Oncology Group (ECOG) score of 0 or 1, and have life expectancy of >3 months. Patients were excluded for inadequate liver function (Grade 2+ ascites, serum albumin < 3.0 g/dL, and total bilirubin > 2.0 mg/dL), radiation exposure to the lungs of > 30 Gy in a single fraction or 50 Gy in multiple administrations, and uncorrectable flow of ^90^Y microspheres to the gastrointestinal tract. In addition, individuals who could not be able to perform pulmonary function tests were not eligible for this study. On the other hand, presence of portal vein thrombus and/or extrahepatic metastasis was not a criterion for exclusion.

### SIRT planning and procedure

All subjects were treated with Yttrium-90 labeled resin microspheres (SIR spheres®, SIRTeX Medical Limited, North Sydney, N.S.W. Australia) on a lobar basis. The details for the procedure can be found in the 2013 version of the SIR-Spheres® product insert and in the report by Kennedy et al. [16, 17]. A pre-treatment diagnostic angiogram was performed in all patients, and specific extrahepatic vessels were coil embolized to prevent ^90^Y-microspheres from being distributed into the visceral organs other than the liver during the SIRT procedure. Arteries that were actively sought and embolized included the gastroduodenal artery, right gastric artery, pancreaticoduodenal vessels, and any other relevant arteries, depending on the patient-specific anatomy. Technetium-99m-labeled macro-aggregated albumin (^99m^Tc-MAA) particles were used for planar imaging and liver-lung shunting calculation according to the EANM (European Association of Nuclear Medicine) guidelines [18]. ^90^Y treatment was contraindicated for patients with a shunt > 20%, while shunts of 11–15% and 16–20% required a reduction in ^90^Y dosage of 20 and 40%, respectively, to decrease the risk of radiation pneumonitis. The prescribed activity of microspheres to be delivered was calculated using empirical method proposed for SIR-Spheres®, which incorporates body surface area (BSA, measured in square meters), tumor volume, and total liver volume into the dose calculation [18]. A post-embolization Bremsstrrahlung SPECT/CT (Single photon emission computed tomography/Computed tomography) scan was performed to confirm the location of microsphere delivery in the treatment area.

The activity that may potentially reach the lung was calculated using the formula [19]:

$$ {A}_{\mathrm{lung}}\left[\mathrm{GBq}\right]={A}_{\mathrm{total}}.L/100 $$

where:

*A*_lung_ = lung activity (GBq)

*A*_total_ = total prescribed activity (GBq)

*L* = lung shunt (%)

The absorbed lung dose (lung exposure dose) given a certain amount of activity shunting from the liver to the lung was calculated using the formula [19]:

$$ {D}_{\mathrm{lung}}\left(\mathrm{Gy}\right)=\left(49670\cdotp {A}_{\mathrm{lung}}\right)/{M}_{\mathrm{lung}} $$

where:

*D*_lung_ = lung dose (Gy)

*A*_lung_ = lung activity (GBq)

*M*_lung_ = mass of the lung (g)

According to the recent prospective autopsy studies, the authors proposed the mean lung weights to be 395 and 445 g for the left and right lungs (total 840 g), respectively, for men and 299 and 340 g (total 639 g), respectively, for women [20, 21]. All tissue densities are estimated at 1 g/mL [19].

The subjects’ data were collected on structured forms by the researchers and were entered into an Excel worksheet which was prepared with these equations.

### DLCO assessments

DLCO tests were performed at baseline; 2, 4, and 8 weeks after the treatment; and after the completion of the treatment. The maximum of the post-treatment values was used for the analyses. DLCO calculations were based on the findings of pulmonary function tests performed using a whole-body plethysmograph (Collins GS II, Collins, Braintree, MA, USA) [22]. Results were compared with reference values based on age, height, gender, and race. The technical specifications of the equipment and the test performance method were made to meet the American Thoracic Society standards for spirometers. The detection of DLCO by a single breath method was repeated at least twice each time, and values less than 5% were made to meet the American Thoracic Society standards. DLCO was corrected to the hemoglobin value measured within 24 h after the test. For the purpose of analysis, an impaired carbon monoxide diffusion capacity is defined as a DLCO value ≤ 80 mmol/min/kPa. Decrease in DLCO is described as a percentage decrease from the pre-RT value (i.e., the percentage decrease in DLCO = (1 − post/pre) × 100).

### Assessment of pulmonary toxicity and radiation pneumonitis

RTOG/EORTC (Radiation Therapy Oncology Group and the European Organization for Research and Treatment of Cancer) Late Radiation Morbidity Scoring Scheme was used to assess the pulmonary toxicity and radiation pneumonia [23]. Patients were monitored for symptoms for a minimum duration of 6 months after SIRT, and a routine PET-CT (Positron emission tomography-Computed tomography) imaging was performed in all patients approximately 75 days after SIRT, particularly focusing on the identification of radiation pneumonitis.

### Statistical analysis

Statistical Package for Social Sciences (SPSS) version 21 was used for the analysis of data. Data are presented as mean ± standard deviation or number (percentage), where appropriate. Normality was tested using both hypothesis tests and graphical methods. The significance of the change between DLCO values before and after the treatments was examined using *t* test for paired samples or Wilcoxon’s Signed Rank test, based on the distribution. Pre-treatment and post-treatment frequencies were compared using McNemar test. The correlations with percent changes in DLCO values were tested using Spearman’s or Pearson’s correlation test, depending on the distribution. A *p* value smaller than 0.05 was considered the indication for statistical significance.

## Results

Patient and clinical characteristics of all patients and the patients that received two treatments are presented in Table 1. Approximately two thirds of the patients were male, and colon cancer was the most frequent indication for treatment constituting half of the cases.

**Table 1:** Patient characteristics

All patients (*n* = 40)	
Age, year	56.9 ± 10.8
Male gender, *n* (%)	28 (70.0%)
Tumor type, *n* (%)	
Colon	20 (50.0%)
Hepatocellular carcinoma	7 (17.5%)
Pancreas Ca	6 (15.0%)
Others*	7 (17.5%)
Lung shunt fraction, % (1st treatment)	6.7 ± 2.6
Lung shunt fraction with 5–10%, *n* (%)	26 (65%)
Lung shunt fraction with 10–15%, *n* (%)	6 (15%)
Lung shunt fraction with 15–20%, *n* (%)	0 (0%)
Treatment radiation dose, GBq (1st treatment)	1.6 ± 0.3
Lung exposure dose, Gy (1st treatment)	6.7 ± 2.8
Patients receiving two treatments (n = 11)	
Age, year	58.5 ± 11.0
Male gender, n (%)	7 (63.6%)
Tumor type, n (%)	
Colon	6 (54.5%)
Hepatocellular carcinoma	2 (18.2%)
Pancreas Ca	1 (9.1%)
Others†	2 (18.2%)
Lung shunt fraction, % (2nd treatment)	7.5 ± 3.0
Lung shunt fraction with 5–10%, *n* (%)	6 (55%)
Lung shunt fraction with 10–15%, *n* (%)	1 (9%)
Lung shunt fraction with 15–20%, *n* (%)	2 (18%)
Treatment radiation dose, GBq (2nd treatment)	1.6 ± 0.2
Lung exposure dose, Gy (2nd treatment)	7.7 ± 3.0

Unless otherwise stated, data are presented as mean ± SD

*Gastric ca (*n* = 2), parotid ca (2), breast ca (1), cholangiocarcinoma (1), unknown (1)

†Cholangiocarcinoma (1), unknown (1)

The mean DLCO value did not significantly change after the first (82.8 mmol/min/kPa ± 19.4 vs. 83.1 ± 20.9, *p* = 0.921) and the second treatments (87.4 ± 19.7 vs. 88.6 ± 23.2, *p* = 0.256). In addition, the frequency of patients with impaired DLCO at baseline did not change significantly after the first (37.5 vs. 45%, *p* = 0.581) and the second treatments (27.3 vs. 27.3%, *p* = 1.000).

**Fig. 1:**
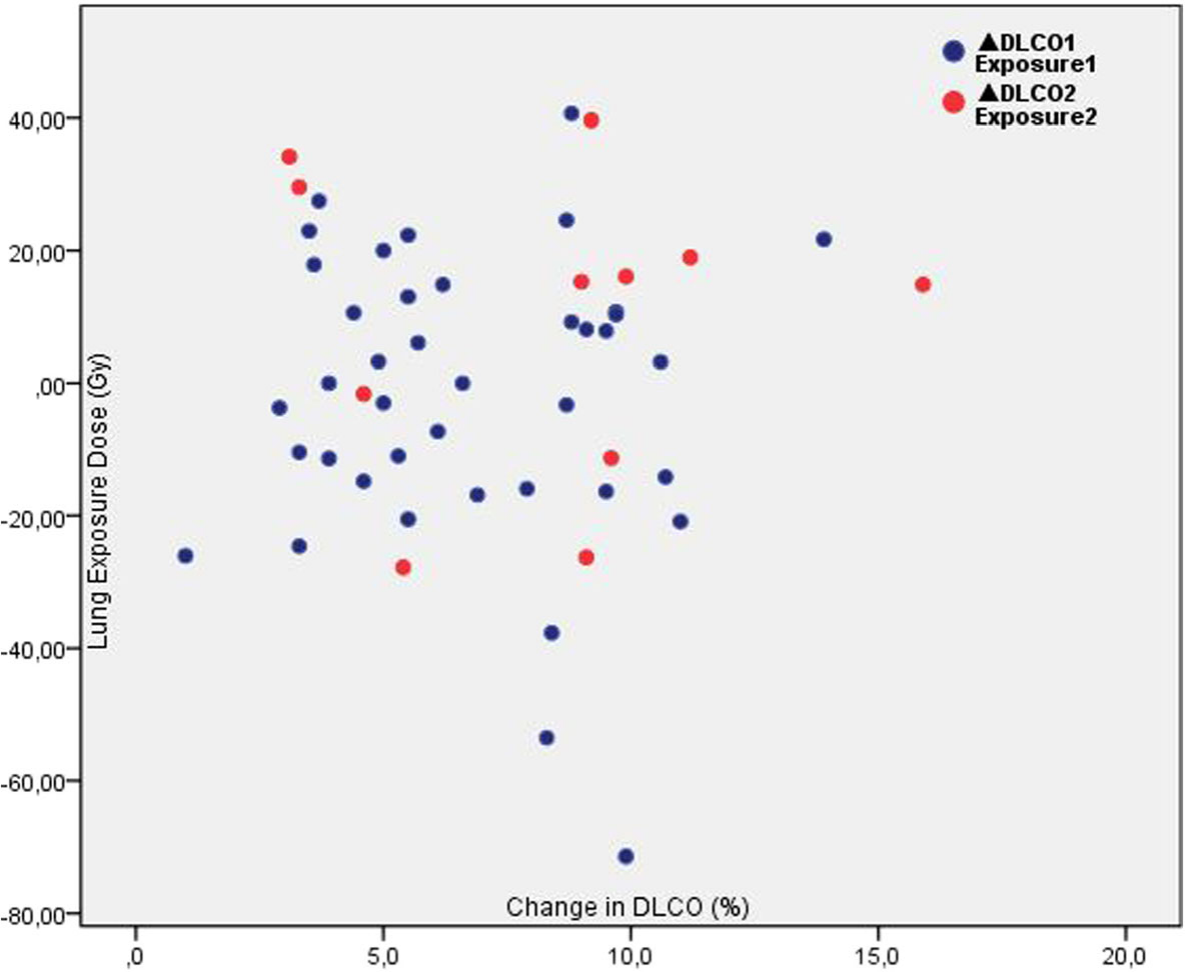
Scatter plot of percent changes in diffusing capacity of the lung for carbon monoxide (DLCO) versus lung exposure dose (Gy)

Percent change in DLCO values (Fig. 1) after the first treatment did not significantly correlate with radiation dose (*r* = 0.221, *p* = 0.170), lung shunt fraction (*r* = − 0.171, *p* = 0.292), or lung exposure dose (*r* = − 0.043, *p* = 0.792). Similarly, these three parameters did not also correlate with DLCO change after the second treatment (*r* = − 0.357, *p* = 0.282; *r* = 0.170, 0.617; and *r* = − 0.029, *p* = 0.933, respectively).

None of the patients developed radiation pneumonitis during follow-up.

## Discussion

To the best of our knowledge, this study is the first to examine the effect of internal dose absorption of the lungs in patients undergoing SIRT using DLCO, which is a reliable indicator of lung function. However, no significant changes in DLCO were found both after the first and second SIRT procedures (Table 2). Furthermore, the percent change in DLCO did not correlate with the treatment radiation dose, lung shunt fraction, or dose exposure in the lungs. Notably, no patients developed pneumonitis during follow-up.

**Table 2:** Changes in lung carbon monoxide diffusion capacity after treatments

	First treatment (*n* = 40)	Second treatment (*n* = 11)
DLCO, mmol/(min/kPa), (pre-treatment)	83.1 ± 20.9	88.6 ± 23.2
DLCO mmol/(min/kPa), (post-treatment)	82.8 ± 19.4	87.4 ± 19.7
% change in DLCO	− 2.2 ± 22.2	− 1.0 ± 20.5
*P* for difference	0.921	0.256
Impaired DLCO, *n* (%) (pre-treatment)	18 (45.0%)	3 (27.3%)
Impaired DLCO, *n* (%) (post-treatment)	15 (37.5%)	3 (27.3%)
*P* for difference	0.581	1.000

Unless otherwise stated, data are presented as mean ± SD

SIRT is an established treatment modality for chemoresistant, unresectable, primary, or metastatic hepatic malignancies. During the development phase of this therapeutic modality, tumor-induced arteriovenous pulmonary shunting and possible occurrence of RP emerged as potential limitations of the technique [2, 4, 24–26]. Post-treatment development of interstitial pneumonia and presence of micro-spheres in the lung biopsy were reported by Lin et al. in 1994 in a hepatocellular carcinoma patient who was treated with resin micro-spheres and who had a pulmonary shunt of 17% [24]. After that, Leung et al. reported the death of three out of five patients developing RP after SIRT [2]. While Salem et al. observed no clinical radiation pneumonitis in a total of 58 patients receiving higher-than-recommended doses (> 30 Gy) [4], Dobrocky et al. published a case report where an asymptomatic patient had radiation pneumonitis within 3 months following treatment as confirmed both by imaging modalities and lung biopsy [26].

An association between deterioration of DLCO and radiation pneumonitis has been reported by many authors. Guerra et al. investigated the possible association between the extent of change in DLCO after external radiotherapy and radiation pneumonitis in 140 patients, based on the hypothesis that patients with symptomatic radiation pneumonitis would experience a significant decrease in pulmonary function that could be quantified using DLCO [27]. These investigators found that patients who experienced higher percent reductions in DLCO were more likely to have high-grade radiation pneumonitis. The main difference between that study and ours lies in the type of radiation exposure, which was internally administered in the latter.

The incidence of radiation pneumonitis may differ between internal vs. external beam radiotherapy as a result of exposure differences. In 46 patients undergoing endobronchial brachytherapy due to malignant airway obstruction, radiation pneumonitis occurred only in those who received external beam therapy [28]. Among 80 subjects who received SIRT-alone, only five developed radiation pneumonitis [2]. In patients receiving external radiotherapy, lung injury has also been reported in non-RT areas, although of lesser severity, and CD4 + lymphocytic alveolitis similar to hypersensitivity pneumonia was described in both lungs, regardless of radiation exposure [29].

Lungs are one of the most radiation sensitive tissues in the body, and among the anatomical components of the lung, alveolar capillary complex exhibits highest sensitivity [30]. Within days to weeks after radiation exposure, initial cytokine release is triggered. Generally, cytokine release occurs within 2 weeks and has no associated symptoms. Second phase of cytokine release starts 6–8 weeks after radiation which is associated with hypoxemia and lung hypoperfusion [31, 32]. In our study, timing of DLCO coincided with this phase to detect the inflammation. The reduction in DLCO is thought to reflect a limited reserve of gas exchange resulting from the potential toxicity of radiotherapy. Although the reduction in DLCO is generally considered to be subclinical, it may provide a sensitive marker of chemotherapy-induced lung injury [12]. In our study, absence of a significant reduction in DLCO may be accounted for by the lower degree of direct exposure to radiation as compared to external beam radiation, leading to minimal injury.

Since radiation pneumonitis was not detected neither clinically nor radiologically during in the follow-up period in our study, we can affirm that the degree of lung injury was not severe in our cohort. However, a comparison with previous cohorts, and particularly with that of Salem et al., shows significantly lower level of radiation exposure in our patients [4], possibly explaining the lack of a statistically significant association between treatment dose, radiation exposure, and the change in DLCO before and after treatment. However, it should be noted that our study was not designed to identify the lowest dose level associated with the development of radiation pneumonitis following SIRT. Nevertheless, our results may give an idea regarding the safe dose range for SIRT.

In this study, no patients who were routinely followed up radiologically with PET/CT imaging at 75 days after SIRT and clinically for 6 months developed any signs or symptoms consistent with radiation pneumonitis. In patients who received a repeated treatment, PET/CT images obtained approximately 150 days after the first treatment also showed no pathology. As pointed out by Dobrocky et al. who used comparable treatment doses, early identification of asymptomatic radiation pneumonitis patients may bear clinical significance [26]. As recommended by Sangro et al., detection of the decline in DLCO values may prove to have clinical utility in these patients, particularly when the high radiation exposure associated with CT scans is taken into consideration [11].

In some patients with hepatic malignancy, repeated doses of SIRT may be required, increasing the likelihood of radiation pneumonitis. About one fourth of the cases were retreated in this study. Furthermore, our study did not show a statistically significant change in DLCO values in association with the SIRT procedure in the second treatment session.

Our study had some significant limitations. Ideally, this study should be repeated with a larger sample size with true lung volumes. In our study, lung mass was not assumed to be 1 kg based on International Commission on Radiological Protection (ICRP). Recent prospective autopsy studies by Molina et al. showed that there was no significant relationship between lung weights, either individually or combined, with body length, body weight, or BMI for healthy adult men (*n* = 232) and women (*n* = 102) [20, 21]. These authors also found no significant difference between the lung weights of underweight, normal weight, overweight, or obese individuals for men and women. The authors, therefore, proposed the mean lung weights to be 395 and 445 g for the left and right lungs (total 840 g), respectively, for men and 299 and 340 g (total 639 g), respectively, for women. Even though this approach lead to higher calculations of absorbed lung doses, we thought it will be more logical. Finally, the results could be different in another study population with higher lung shunt fractions, e.g., in patients with hepatocellular carcinoma with major vascular invasion.

Recently, Sancho et al. showed that imaging with ^99m^Tc-MAA is essential in SIRT workup in their retrospective analysis of 532 consecutive patients [33]. However, in contrast to the study by Jha et al., some researchers have reported that planar lung shunt fractions were overestimated with ^99m^Tc-MAA scans [34–39]. There were several explanations for this result. First, there was a correlation between scan quality and lung shunt fraction, suggesting that low scan quality leads to overestimation [40]. Second, according to O’Doherty et al., the scatter correction should be used on pretreatment ^99m^Tc-MAA scans in order to more accurately assess the lung shunting percentage before therapy [41]. Lastly, tracer degradation leads to overestimation of lung shunt fraction [42]. Since no cases developed RP in this study and DLCO values did not change significantly after the SIRT procedure, we can predict that a possible overestimation of the absorbed lung dose will not change our results. Larger studies with comparable protocols (planar vs. SPECT/CT images, ^99m^Tc-MAA scans vs. ^90^Y post-radioembolization images, ^99m^Tc-MAA scans vs. ^90^Y-microsphere PET/CT images, etc.) are required to better delineate DLCO changes after SIRT, especially in patients with higher lung shunt fractions.

The association between the alterations in DLCO and the severity of potential radiation pneumonitis developing after SIRT may prove to be clinically important. Since no cases developed radiation pneumonitis in this study, a cutoff value for the decline in DLCO could not be defined. If the assumed relationship between the percent change in DLCO and severity of radiation pneumonitis proves to be valid, then it may be possible to identify potential candidates for radiation pneumonitis using DLCO monitoring.

## Conclusions

Findings of this study do not suggest a significant change in DLCO values in association with the SIRT procedure, either at the first or the second treatment sessions. Further large studies possibly with different protocols are warranted to better delineate DLCO changes after SIRT in a larger spectrum of patients.

## Abbreviations

^90^Y: Yttrium-90
^99m^Tc-MAA: Technetium-99m-labeled macro-aggregated albumin
BSA: Body surface area
CT: Computed tomography
DLCO: Diffusion capacity of the lungs for carbon monoxide
EANM: European Association of Nuclear Medicine
ECOG: Eastern Cooperative Oncology Group
ICRP: International Commission on Radiological Protection
PET: Positron emission tomography
RP: Radiation induced pneumonitis
RT: External beam radiotherapy
RTOG/EORTC: Radiation Therapy Oncology Group and the European Organization for Research and Treatment of Cancer
SIRT: Selective internal radiation therapy
SPECT: Single photon emission computed tomography
SPSS: Statistical Package for Social Sciences
